# Identification of immune microenvironment subtypes and signature genes for Alzheimer’s disease diagnosis and risk prediction based on explainable machine learning

**DOI:** 10.3389/fimmu.2022.1046410

**Published:** 2022-12-08

**Authors:** Yongxing Lai, Peiqiang Lin, Fan Lin, Manli Chen, Chunjin Lin, Xing Lin, Lijuan Wu, Mouwei Zheng, Jianhao Chen

**Affiliations:** ^1^ Department of Geriatric Medicine, Shengli Clinical Medical College of Fujian Medical University, Fujian Provincial Hospital, Fuzhou, Fujian, China; ^2^ Fujian Provincial Center for Geriatrics, Fujian Provincial Hospital, Fuzhou, Fujian, China; ^3^ Department of Neurology, Shengli Clinical Medical College of Fujian Medical University, Fujian Provincial Hospital, Fuzhou, Fujian, China; ^4^ Department of Neurology, Fujian Medical University Union Hospital, Fuzhou, Fujian, China; ^5^ Department of Rehabilitation Medicine, Shengli Clinical Medical College of Fujian Medical University, Fujian Provincial Hospital, Fuzhou, Fujian, China

**Keywords:** alzheimer’s disease, immune microenvironment, characteristic genes, machine learning, immune subtypes

## Abstract

**Background:**

Using interpretable machine learning, we sought to define the immune microenvironment subtypes and distinctive genes in AD.

**Methods:**

ssGSEA, LASSO regression, and WGCNA algorithms were used to evaluate immune state in AD patients. To predict the fate of AD and identify distinctive genes, six machine learning algorithms were developed. The output of machine learning models was interpreted using the SHAP and LIME algorithms. For external validation, four separate GEO databases were used. We estimated the subgroups of the immunological microenvironment using unsupervised clustering. Further research was done on the variations in immunological microenvironment, enhanced functions and pathways, and therapeutic medicines between these subtypes. Finally, the expression of characteristic genes was verified using the AlzData and pan-cancer databases and RT-PCR analysis.

**Results:**

It was determined that AD is connected to changes in the immunological microenvironment. WGCNA revealed 31 potential immune genes, of which the greenyellow and blue modules were shown to be most associated with infiltrated immune cells. In the testing set, the XGBoost algorithm had the best performance with an AUC of 0.86 and a P-R value of 0.83. Following the screening of the testing set by machine learning algorithms and the verification of independent datasets, five genes (CXCR4, PPP3R1, HSP90AB1, CXCL10, and S100A12) that were closely associated with AD pathological biomarkers and allowed for the accurate prediction of AD progression were found to be immune microenvironment-related genes. The feature gene-based nomogram may provide clinical advantages to patients. Two immune microenvironment subgroups for AD patients were identified, subtype2 was linked to a metabolic phenotype, subtype1 belonged to the immune-active kind. MK-866 and arachidonyltrifluoromethane were identified as the top treatment agents for subtypes 1 and 2, respectively. These five distinguishing genes were found to be intimately linked to the development of the disease, according to the Alzdata database, pan-cancer research, and RT-PCR analysis.

**Conclusion:**

The hub genes associated with the immune microenvironment that are most strongly associated with the progression of pathology in AD are CXCR4, PPP3R1, HSP90AB1, CXCL10, and S100A12. The hypothesized molecular subgroups might offer novel perceptions for individualized AD treatment.

## Introduction

Alzheimer’s disease (AD) is a severe progressive neurodegenerative disease characterized by the over-accumulation of amyloid-beta (Abeta) plaques, neurological deficits, and cognitive impairment (1). Around 2-8% (more than 50 million) of the world’s population has been impacted by AD over the past few decades, placing a significant medical cost on society (2). Only a select group of medications, including acetylcholinesterase inhibitors and N-methyl-D-aspartate antagonists, have been given FDA approval to treat cognitive impairment in AD patients (3). Unfortunately, the clinical complexity and personal heterogeneity of AD patients, in addition to the severe side effects, may compromise the effectiveness of pharmacological treatment (4). In addition, most patients lose the best opportunity for treatment after initial diagnosis because of AD’s gradual and progressive onset. Therefore, it is essential to identify reliable diagnostic markers for the early diagnosis of AD and to develop novel molecular stratification techniques to direct the customized treatment of AD patients.

Recent research has shown that changes to peripheral and central immune cells may exert crucial roles in accelerating the progression of AD (5, 6). For instance, peripheral B lymphocytes, an essential component of adaptive immune systems, can enter the central nervous system of AD patients, breach the blood-brain barrier, and promote the activation of the immune response through interactions with resident brain cells (6). It has been reported that type 1 and type 17 T cells, two subclasses of CD4+ T cells, contributed to the development of AD by triggering glial pro-inflammatory responses (7). In addition, the initiation of the innate immune system is strongly linked to the beginning of AD. It has been suggested that natural killer cells, which are known for their capacity to destroy infected cells, may increase the risk of AD damage by mediating Aβ-dependent cytotoxicity (8). Another important pathogenic mechanism contributing to the poor prognosis of neuroinflammatory and neurodegenerative illnesses is the release of harmful mediators from mast cells (9). In AD patients, changes in peripheral dendritic cells are linked to severe clinical symptoms (10). Moreover, the increased neutrophils is more likely to contribute to excessive accumulation of Aβ and the impairment of memory and cognitive abilities (11). These findings underline the immune system’s crucial function in AD. Therefore, to identify AD patients who may benefit from immunotherapy, detailed research of immune microenvironment-related characteristic genes and precise identification of immune molecular subtypes are urgently required.

We set out to evaluate the immune microenvironment patterns in AD patients in great detail in this work. To identify differentially expressed immune cells, the ssGSEA and LASSO algorithms were used. Several machine learning algorithms, such as Light Gradient Boosting (LightGBM), CatBoost, eXtreme Gradient Boosting (XGBoost), Random Forest (RF), Logistic Regression (LR), and support vector machines (SVM), were developed to predict AD outcomes and identify characteristic genes associated with immune microenvironment. For analyzing the results of machine learning models, the SHAP and LIME algorithms were used. For the purpose of estimating the relationship between these distinctive genes and AD pathology biomarkers, correlation analysis was carried out. In addition, we identified distinct subtypes of immunological microenvironments based on the expression of distinguishing genes. We also looked at how these subgroups varied in terms of enhanced functions, pathways, immune cell infiltration, immunological features, and therapeutic medicines. Finally, to further validate the expression of distinctive genes, RT-PCR analysis, pan-cancer analysis, and another online database called AlzData were used.

## Materials

### Raw data acquisition and preprocessing

**Figure 1:**
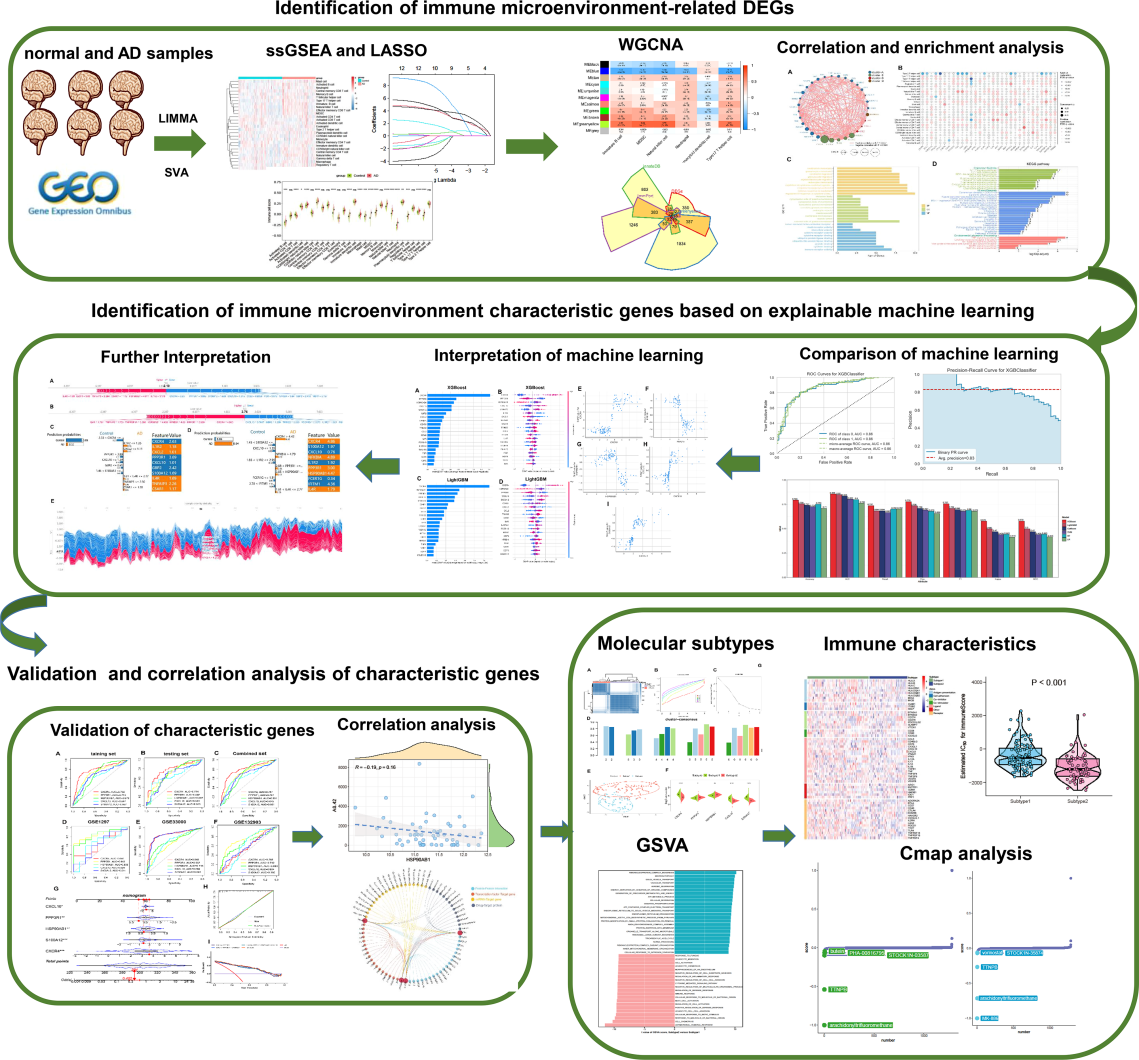
The study flow chart. **Step1:** Identification of immune microenvironment-related DEGs. 1) AD-related immune cells shared by the integrated and external validation datasets were found using the LASSO and ssGSEA algorithms. 2) The immune microenvironment-related DEGs were identified using the WGCNA method and immune-related internet resources. 3) Analysis of DEGs connected to the immunological microenvironment in terms of correlation and functional enrichment. **Step2:** Identification of immune microenvironment characteristic genes based on explainable machine learning. 1) Comparison of the diagnostic efficacy of six machine learning algorithms including LightGBM, CatBoost, XGBoost, RF, LR, and SVM. 2) SHAP summary plot SHAP dependency analysis were employed to interpretation the XGBoost and LightGBM models and screen the final characteristic immune genes. 3) The SHAP force plots and the LIME algorithm were applied for interpreting the individualized prediction of AD in the XGBoost model. **Step3:** Validation and correlation analysis of characteristic genes. 1) The adequate diagnostic capacity of these 5 distinctive genes was demonstrated using the external validation datasets, generated nomogram, calibration curve, and decision curve analysis. 2) Correlation analysis between 5 characteristic genes and AD pathological biomarkers including α-secretase, β-secretase, γ-secretase, and Aβ-42 levels. **Step4:** Identification of immune microenvironment-related molecular subtypes in AD patients. 1) Based on the expression profiles of 5 distinctive genes, consensus clustering technique was used to identify AD-related molecular subtypes, and functional enrichment was assessed using GSVA approach. 2) Comparison the immune characteristics between different subtypes. 3) The CMap approach was used to predict prospective therapeutic medicines that would target certain subtypes.

The transcriptome data were obtained from the Gene Expression Omnibus database (GEO, https://www.ncbi.nlm.nih.gov/geo/). The obtained datasets were as follows: GSE5281 (87 AD and 74 normal brain tissues) (12), GSE28146 (22 AD and 8 normal brain tissues) (13), GSE48350 (80 AD and 173 normal brain tissues) (14), GSE122063 (56 AD and 44 normal brain tissues) (15), GSE33000 (310 AD and 157 normal brain tissues) (16), GSE1297 (22 AD and 9 normal brain tissues) (17), GSE132903 (97 AD and 98 normal brain tissues) (18), GSE106241 (60 AD brain tissues). Three GPL570 datasets GSE5281, GSE28146, and GSE48350 were combined on the basis of the Combat function of “sva” R package (19), which finally yielded 247 normal and 189 AD brain tissues after excluding eight abnormal samples, were selected as test sets. Other platforms datasets GSE122063, GSE33000, GSE132903, and GSE106241were selected as the validation sets. The raw data from these GEO datasets were pre-processed and normalized on the basis of the Robust Multiarray Average (RMA) function of “affy” R package. The detailed flow chart of study process was exhibited in **Figure 1**.

### Evaluation of immune infiltrating cells

The enrichment scores of 28 immune cell subtypes were evaluated using the single sample gene set enrichment analysis (ssGSEA) as previously reported (20). Briefly, the overall marker genes were utilized to estimate the abundance of immune cells in a single sample, and the relative proportion of each cell subtype of immune cells was exhibited as an enrichment score. The Wilcoxon rank sum test was applied for evaluating the differences in immune cell proportions between different groups. To estimate the immune infiltrated levels, the immune scores were calculated using the R package “ESTIMATE”. A p-value less than 0.05 was considered statistically different.

### Identification of characteristic immune cells

The least absolute shrinkage and selection operator (LASSO) is a widely used linear regression based on the R package of “glmnet” for high-dimensional data (21, 22). In this study, we conducted the LASSO regression model to determine the optimal variables from immune cells. The combined dataset was randomly divided into the training cohort (70%) and validation cohort (30%), A ten-fold cross-validation method with 1,000 iterations was employed to avoid the underlying instability of the results. The optimal penalty parameter (λ) was determined *via* the minimum criteria. The immune cell subsets with non-zero coefficients were considered the optimal variables and were applied for subsequent analyses.

### Weighted correlation network analysis (WGCNA)

The WGCNA network was established to identify gene modules associated with immune cell subtypes based on the R package of “WGCNA” (23). Briefly, the top 25% of genes with high variance from the combined dataset were selected as the input data to increase the accuracy of the results. The optimal soft threshold power was identified based on the scale-free topology criterion, followed by the construction of a weighted adjacency matrix and the transformation of a topological overlap matrix (TOM). Modules with >50 genes were screened based on the hierarchical clustering tree method. Each module was displayed in a random color. Module eigengene (ME) represented the first principal component of a gene module, and module significance (MS) was represented by the correlation coefficient between modules and corresponding clinical traits. Gene significance (GS) was defined as the relationship between each gene with a clinical phenotype.

### Evaluation of immune microenvironment-associated differential genes

A total of 2483 immune-related genes were obtained from the Immunology Database and Analysis Portal (ImmPort; https://www.immport.org/home), and 1379 immune genes were downloaded from the innateDB (https://www.innatedb.ca/) database. Differentially expressed genes (DEGs) between control and AD samples were identified using the “limma” R package (24), and the criteria of | log2 (fold change) | > 0.5 and a false discovery rate (FDR) of 0.05. Finally, the immune microenvironment-associated DEGs were determined by intersecting the immune-related genes, DEGs, and genes in feature modules.

### Functional enrichment analysis

Gene ontology (GO) biological functions and Kyoto Encyclopedia of Genes and Genomes (KEGG) pathway enrichment analysis were conducted using the R package of “clusterProfiler” (25). GO categories cover biological processes (BP), molecular functions (MF), and cellular components (CC). The p-value was adjusted based on the Benjamini–Hochberg method, and a value of p.adjust less than 0.05 was considered statistically different.

### Machine learning models for feature selection and visualization

Stable and remarkable features are critical for predicting the risk of AD onset and progression. Based on the expression profiles of immune microenvironment-associated DEGs, we applied the PyCaret (3.0.0) Python package for establishing six machine learning models including LightGBM, CatBoost, XGBoost, RF, LR, and SVM. The LightGBM, CatBoost, and XGBoost classifiers are the optimized distributed gradient boosting algorithms with satisfactory predictive efficacy by converting a series of weak variables into strong variables (26–28). The RF algorithm is one of the most acceptable and well-known multi-class tree algorithms that combines decision trees through majority voting, eventually exhibiting its high accuracy and fast independent learning on distinct datasets (29). The LR algorithm is one of the most classic linear prediction algorithms based on the regression coefficients, and has been widely utilized in various fields in recent decades (30). The SVM algorithm is a popular machine learning algorithm that projects input data into a higher-dimensional feature space by mapping a kernel function that is easier to classify than the original feature space. The iterative learning process of the SVM eventually converges to the optimal hyperplane that provides the largest inter-class span (31). These machine-learning models were constructed in accordance with our previous study (32). Briefly, the classification of diseases was recognized as the response variable, and the immune microenvironment-associated DEGs were selected as the explanatory variables. All samples enrolled in the combined dataset were randomly split into a training set (70%) and a validation set (30%). Predictive performance for these machine learning models was estimated using the accuracy, precision-recall (P-R) value, area under the receiving operating characteristic curve (AUC), recall, precision, F1, kappa, and Matthews correlation coefficient (MCC). The final candidate model was determined on the basis of accuracy, AUC, and P-R value.

Subsequently, the Shapley Additive exPlanation (SHAP) values were utilized to visualize key features affecting AD onset and progression, thus analyzing the significance of individual features that influence the prediction of the outcome and exhibiting the impact of each vital feature on the final machine learning model. In addition, we conducted the other explainable algorithm, LIME, to fit the predictive behavior of the optimal machine learning model (33).

### External validation of characteristic genes

External datasets, including GSE33000, GSE1297, and GSE132903 were utilized to verify the ability of immune microenvironment-associated feature genes to distinguish AD from non-AD control, and the diagnostic efficacy was visualized using the AUC curves based on the R package of “pROC”. GSE10624 was utilized to explore the correlation between feature genes and classical pathological markers in AD patients. The mRNA expression data of pan-cancer were downloaded from Genomic Data Commons (GDC, https://gdc.cancer.gov/) and employed to verify the expression levels of characteristic genes in pan-cancer tissues. The AlzData database (http://www.alzdata.org/) was used to depict the expression of characteristic genes in multiple brain tissues of AD patients.

### Establishment of a nomogram

Immune microenvironment-associated feature genes were fitted to establish a nomogram on the basis of the R package of “rms”. The calibration curve was used to figure out how accurate the nomogram was, and the decision curve analysis (DCA) was used to figure out how useful the nomogram was in the clinic.

### Recognition of distinct immune microenvironment subtypes by unsupervised clustering

We performed the “partitioning around medoid” (PAM) method to identify immune microenvironment subtypes for AD patients, based on the ConsensusClusterPlus package. Performance of the cumulative distribution function (CDF) curve, consensus matrix, relative alterations in area under CDF curve, and a consistent cluster score (>0.9) were considered when selecting the optimal subtype numbers. The distribution differences in immune microenvironment subtypes were visualized using a t-Distributed Stochastic Neighbor Embedding (tSNE) plot based on the Rtsne package.

### Gene set enrichment analysis (GSVA)

Functional enrichment between immune microenvironment subtypes was evaluated using the “GSVA” and “limma” packages (34). The gene sets “c2.cp.kegg.v7.4.symbols” and “c5.go.bp.v7.5.1.symbols” were obtained from the Molecular Signatures Database (MSigDB) (https://www.gsea-msigdb.org/gsea/msigdb/). The absolute t-value of the GSVA score of hallmark pathways and biological functions more than 5 were considered statistically different.

### Prediction of small-molecule compounds

Connectivity map (CMap) analysis was conducted to predict small-molecule compounds targeting immune microenvironment subtype 1 and subtype2 as previously reported (35). Briefly, a total of 1309 drug signatures were downloaded from the Connectivity Map database (CMap, https://clue.io/), the expression profiles of the top 150 up-regulated and 150 down-regulated were selected as the input data. The CMap score were calculated using the eXtreme Sum (XSum) algorithm, and the top five small-molecule compounds with the lowest CMap score were selected for visualization.

### Primary culture of cortical neurons

Primary culture of cortical neurons was conducted following our previous study (36) with minor modifications. Briefly, embryonic rats (16-18 days) were dissected from anesthetized pregnant Sprague-Dawley rats (purchased from the Experimental Animal Center of Fujian Medical University). The cerebral cortex of embryonic rats was dissected, and the meninges and blood vessels were carefully exposed. The isolated tissue was then minced, trypsinized with 0.25% trypsin for 20 min at 37°C, and minced gently. Dispersed cells (3 × 10^6^ cells) were placed on 6-well plates coated with poly-1-lysine (100 µg/ml). Subsequently, primary cortical neurons were cultured in a neurobasal medium (Gibco, NY, USA) supplemented with 2% B27 supplement (Gibco, NY, USA), 0.5-mM L-glutamine (Gibco, NY, USA), and 50 U/ml of penicillin-streptomycin (Gibco, NY, USA). The cultured media were first changed after 8 hours and subsequently half of the medium was replaced every 2-3 days. Cortical neurons were cultured for approximately 7-9 days in a 37°C, 5% CO2 incubator. The Institutional Animal Care and Use Committee of Fujian Medical University approved this study, and it was done according to the Guidelines for the Care and Use of Laboratory Animals.

### Oligomeric Aβ preparation and establishment of AD model in primary cortical neurons

The preparation of Aβ_1-42_ oligomer form was conducted following the previously reported (37) with minor modifications. Briefly, a total of 1mg Aβ_1-42_ was dissolved in pre-cooled hexafluoroisopropanol (HFIP) and incubated at room temperature for 30 to 60 minutes, making the concentration of Aβ1-42 was 1mmol/L. Subsequently, the 1mg Aβ_1-42_-HFP solution was placed on the ice for 5-10 minutes, the Aβ_1-42_ peptide membrane was obtained after removing the supernatant and was stored at -20°C. Next, The Aβ_1-42_ peptide membrane was dissolved in DMSO (Gibco, NY, USA) and stored at -20°C until use. To maintain oligomerization conditions, the F-12 medium (Gibco, NY, USA) was added to Aβ_1-42_ peptide membrane and incubated at 4°C overnight. After centrifugation and removal of the precipitation, The supernatant (Aβ_1-42_ peptide) was obtained and utilized to further study. The primary cortical neurons (DIV 7-8) cultured on the 6-well plates were incubated with 20 umol/L Aβ_1-42_ oligomer at 37°C for 12h to mimic the *in vitro* model of AD. The culture medium was then changed to the normal neurobasal medium, and the cells were grown in a 37°C incubator with 5% CO_2_.

### Real-time RT-PCR analysis

The total RNA of primary cortical neurons (DIV 7-8) cultured on the 6-well plates was extracted using a TRIzol reagent (ThermoFisher Scientific, MA, USA) following the manufacturer’s instructions. Total RNA was reverse-transcribed using a RevertAid First Strand cDNA Synthesis Kit (Thermo Fisher Scientific, MA, USA) for complementary DNA (cDNA) synthesis. The primers applied for RT-PCR analysis were as follows: CXCR4: forward, 5′-GTTCCAGTTCCAGCACATCAT-3′, reverse, 5′- CCAGGATAAGGATGACCGTAGT-3’; CXCL10: forward, 5′-TGCAAGTCTATCCTGTCCGC-3′, reverse, 5′-TCTTTGGCTCACCGCTTTCA-3’; PPP3R1: forward, 5′-AAGATACGCAGTTACAGCAGATTG-3′, reverse, 5′-CCACCTACAACAGCACAGAAC-3’; HSP90AB1: forward, 5′-TCTAATGCTTCTGATGCCCTGG′, reverse, 5′-GTGTCCACCAAAGTCAGCGT-3’; S100A12: forward, 5′-CTTCCACCAATACTCAGTTCGG′, reverse, 5′-GCAATGGCTACCAGGGATATG-3’. The qRT-PCR was performed with SYBR^®^ Premix Ex Taq™ II (Takara, Shiga, Japan) and an ABI 7500 Real-Time PCR system (Applied Biosystems, CA, USA). Relative quantification was conducted against a standard curve, and the specific values were normalized against rat β-actin mRNA. The results were defined as a relative increment in mRNA expression relative to control values.

### Other statistical analysis

All statistical analyses were performed using R software (version 4.1.0). The correlation analysis was performed on the basis of the Spearman method. The Wilcoxon sum-rank test was utilized to compare the difference between two groups. The FDR was calculated by the Benjamin–Hochberg method for adjusting the p-value. Statistical significance was defined as a two-sided p < 0.05.

## Results

### Infiltration of immune cells results in AD patients

**Figure 2:**
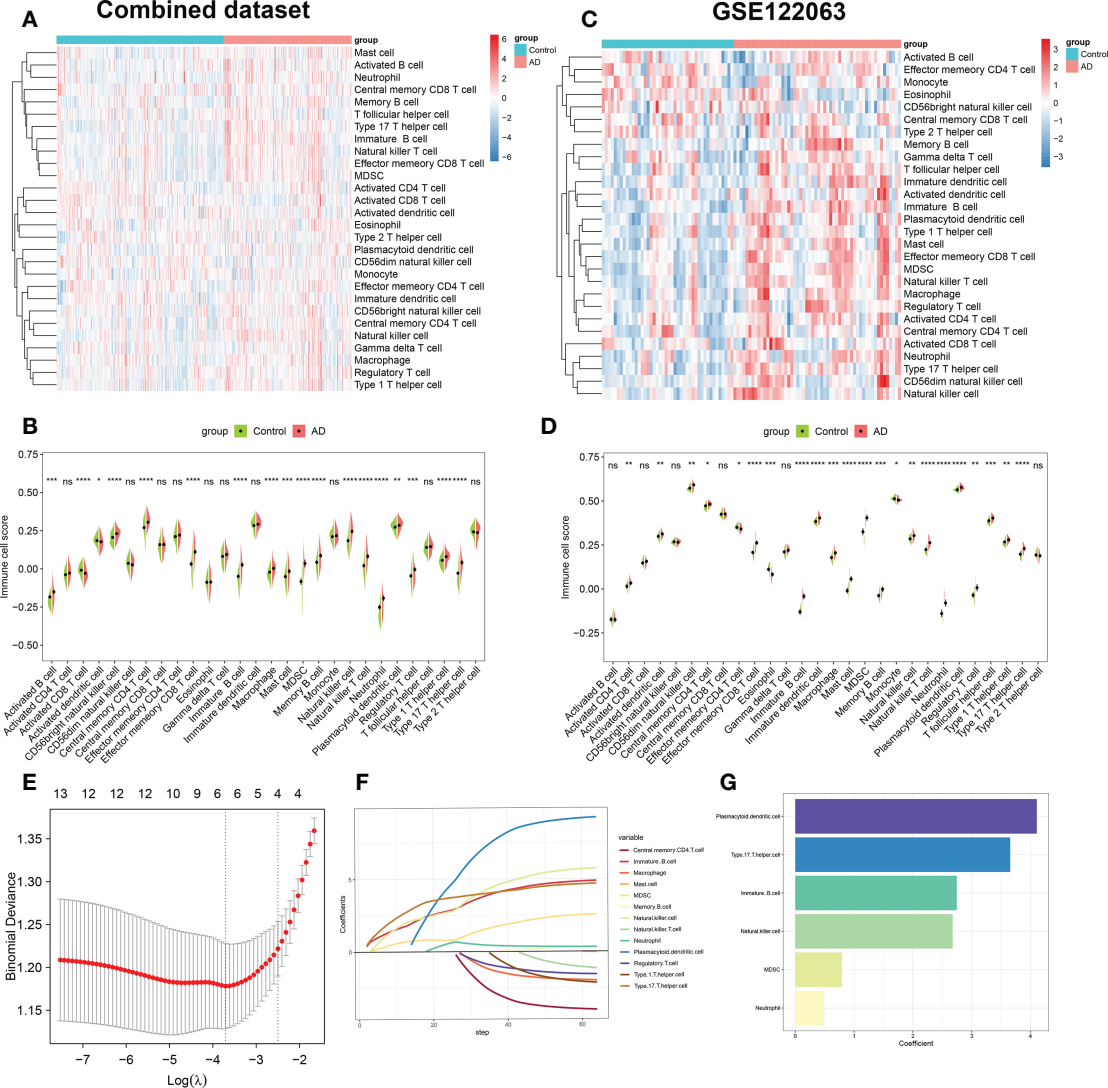
Evaluation of immune cell infiltration between AD and non-AD individuals. **(A)** Heatmap showing the ssGSEA scores of 28 immune cell subpopulations in combine dataset. **(B)** Violin plot showing the differences of infiltrated immune cells in combine dataset between AD and normal individuals. *p < 0.05, **p < 0.01, ***p < 0.001, ****p < 0.0001, ns, no significance. **(C)** Heatmap showing the ssGSEA scores of 28 immune cell subpopulations in GSE122063. **(D)** Violin diagram showing the differences of infiltrated immune cells in GSE122063 between AD and normal individuals. *p < 0.05, **p < 0.01, ***p < 0.001, ****p < 0.0001, ns, no significance. **(E)** Ten-fold cross-validation of LASSO regression analysis. Error bars represented the standard error (SE). The dotted vertical lines corresponded to the optimal value of lambda. **(F)** LASSO coefficient profiles of 13 differentially expressed immune cells. **(G)** Barplots showing six immune cells with non-zero coefficients recognized by LASSO algorithm.

We first combined the normal and AD brain tissue expression profiles of the GSE48350, GSE5281, and GSE28146 datasets, and after excluding 8 abnormal brain tissues, we obtained 247 normal brain tissues and 189 AD brain tissues. Before removing batch effects, brain tissues from different platforms showed significantly different clustering patterns but grouped together after batch correlation (**Figure S1**). To characterize immune differences between non-AD controls and AD patients, we compared the difference in enrichment scores of 28 immune cell subsets in each group of the combined dataset. We observed a higher B cell infiltration, including that of activated B cell, immature B cell, and memory B cell in patients with AD. Meanwhile, AD patients exhibited higher T cell scores, including the central memory CD4^+^ T cell, the effector memory CD8^+^ T cell, the natural killer T cell, the regulatory T cell, the type 1 T helper cell, and the type 17 T helper cell. In addition, natural killer cell, macrophage, mast cell, MDSC, neutrophil, and dendritic cell also had higher cell scores in patients with AD (**Figures 2A, B**). To further validate the results of immune infiltration, we next evaluated the differences in the 28 immune cell scores in GSE122063. The results revealed that excepting activated B cell, activated CD8+ T cell, CD56bright natural killer cell, central memory CD8+ T cell, gamma delta T cell, type 2 T helper cell, the remaining 22 immune cell enrichment scores displayed notable differences between the control and AD groups (**Figures 2C, D**).Combining these results, we finally identified 13 differentially expressed immune cells in AD patients including central memory CD4 T cell, effector memory CD8^+^ T cell, immature B cell, macrophage, mast cell, MDSC, memory B cell, natural killer cell, natural killer T cell, neutrophil, Plasmacytoid dendritic cell, regulatory T cell, type 1 T helper cell, and type 17 T helper cell, suggesting that the alterations in infiltrated immune cells are closely linked to AD pathology.

Subsequently, we performed the LASSO regression algorithm to further determine the characteristic immune cells related to the progression of AD. Six optimal variables (plasmacytoid dendritic cell, type 17 T helper cell, immature B cell, natural killer cell, MDSC, and neutrophil) with non-zero coefficients were finally determined from the above 13 immune cell subsets (**Figures 2E–G**).

### Identification of immune microenvironment-related DEGs

**Figure 3:**
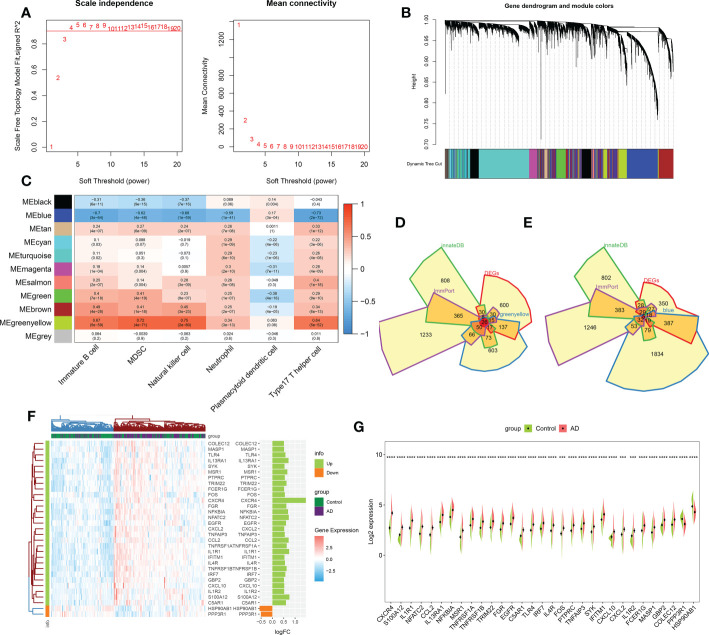
Identification of immune microenvironment-related DEGs between AD and controls. **(A)** The selection of soft threshold β. **(B)** Dendrogram of the co-expression module clustering tree. Distinct colors corresponded to the different co-expression modules. **(C)** Heatmap indicating the correlation between 11 modules with six types of immune cell. **(D)** Venn diagram showing the immune microenvironment-related DEGs shared by greenyellow module (986 genes), DEGs of AD (863 genes), immune genes from ImmPort (2483 genes) and innateDB (1879 genes) databases. **(E)** Venn diagram indicating the immune microenvironment-related DEGs shared by blue module (2426 genes), DEGs of AD (863 genes), immune genes from ImmPort (2483 genes) and innateDB (1879 genes) databases. **(F, G)** Heatmap **(F)** and split violin plots **(G)** revealing the expression of 31 immune microenvironment-related DEGs between AD and healthy controls. ***p < 0.001, ****p < 0.0001.

We performed the WGCNA on the basis of the expression profile of the combined dataset to determine core modules correlated with the above six characteristic immune cell subtypes in patients with AD. A scale-free topology network and connectivity were most efficient when the soft threshold β was set at 4 based on the PickSoftThreshold function (**Figure 3A**). The clustering tree was classified into eleven differently colored gene modules *via* a hierarchical clustering algorithm (**Figure 3B**). Among these modules, the greenyellow module (986 genes) had the highest positive correlation with immature B cell (R = 0.67), MDSC (R= 0.72), natural killer cell (R= 0.75), neutrophil (R= 0.34), and type17 T helper cell (R= 0.64). Whereas the blue module (2426 genes) exhibited the highest negative correlation with immature B cell (R = -0.7), MDSC (R= -0.62), natural killer cell (R= -0.68), neutrophil (R= -0.59), and type17 T helper cell (R= -0.73) (**Figure 3C**). Therefore, we selected the genes inside the greenyellow and blue modules for further analysis. Following intersection, 26 immune microenvironment-related DEGs shared by greenyellow module-related genes, DEGs of AD, immune-related genes from the ImmPort and innateDB datasets were finally identified (**Figure 3D**). In addition, we also determined 5 immune microenvironment-related DEGs in the blue module (**Figure 3E**). Further, a unique immune microenvironment profile was generated between control and AD groups when compared to the expression levels of these 31 immune microenvironment-related DEGs. HSP90AB1 and PPP3R1 expression levels were significantly lower in AD patients than in non-AD controls, whereas the remaining 29 DEGs were significantly higher in AD patients (**Figures 3F, G**), suggesting that these immune microenvironment-related DEGs may be closely linked to AD progression.

### Correlation and functional enrichment analysis of immune microenvironment-related DEGs

**Figure 4:**
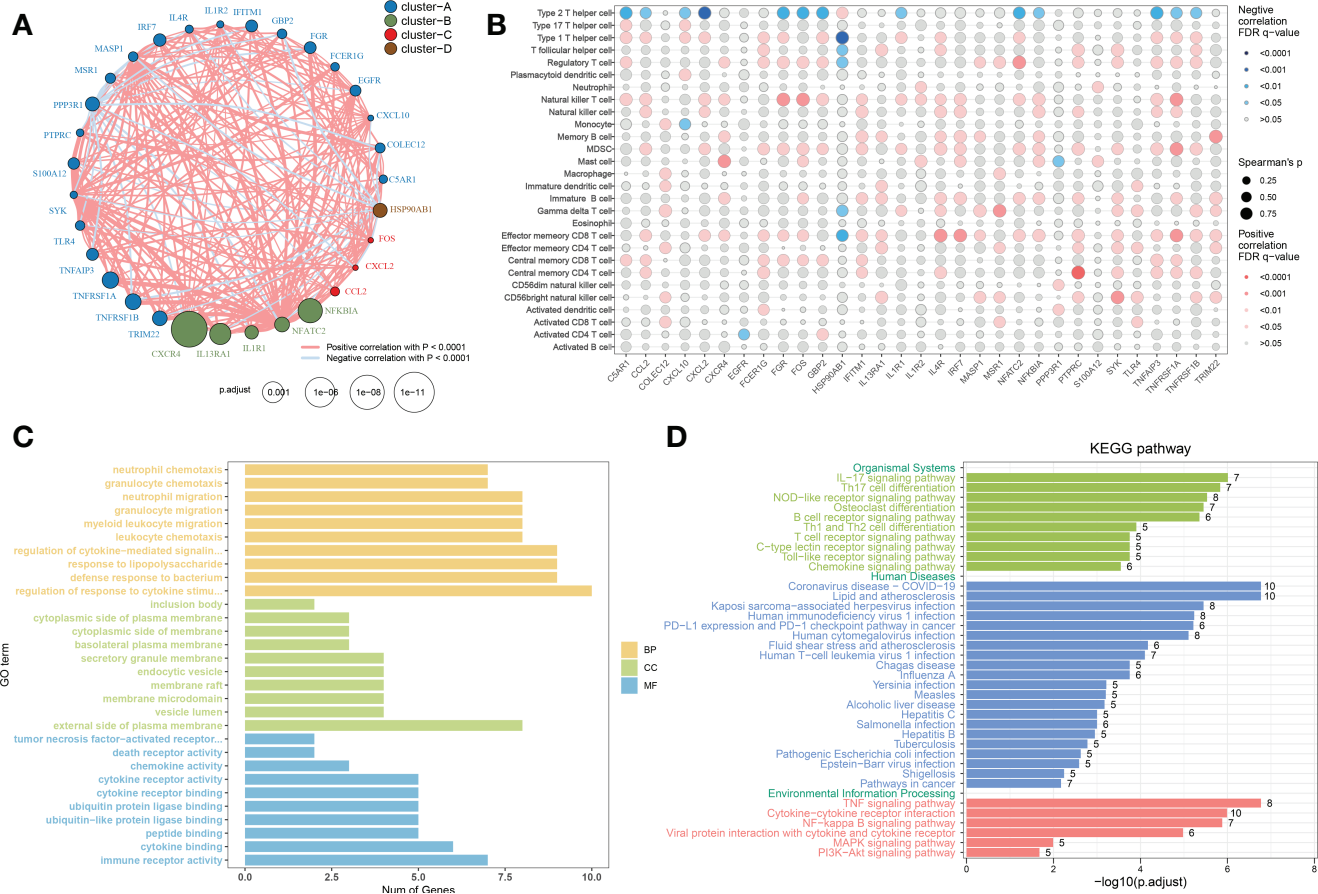
Correlation and functional enrichment analysis of immune microenvironment-related DEGs. **(A)** Interactions among 31 immune microenvironment-related DEGs. The circle size represented the impact of each regulator on AD, the p-value was adjusted based on the Benjamini–Hochberg method. Different colors corresponded to different gene clusters. The lines connecting the DEG represented interactions, and the thickness of the lines represented the strength of the correlation. Red color corresponded to positive correlations and blue color corresponded to negative correlations. **(B)** The correlation between 31 immune microenvironment-related DEGs and 28 immune cell subsets. The circle size represented the value of correlation coefficients. Red color corresponded to positive correlations and blue color corresponded to negative correlations. The shade of color represented the adjusted p-value. **(C, D)** Barplots showing the GO **(C)** and KEGG **(D)** enrichment analysis of 31 immune microenvironment-related DEGs.

To evaluate the correlation among immune microenvironment-related DEGs, we first depicted the comprehensive landscape of 31 immune microenvironment-related DEGs interactions based on the gene expression data and identified four distinct patterns. Among these DEGs, most of which exhibited strong synergistic effects (**Figure 4A**). In addition, we depicted the correlation patterns between 31 immune microenvironment-related DEGs and 28 immune cell subsets. Consistently, the results underlined that these immune microenvironment-related DEGs were significantly correlated with immune cells (**Figure 4B**), indicating the alterations in immune microenvironment may be a vital pathophysiological mechanism contributing to AD progression. Functional enrichment analysis revealed that these immune microenvironment-related DEGs were mainly enriched in biological functions including immune responses, cytokine–cytokine receptor interaction, regulation of plasma membrane, ubiquitin protein ligase binding, and TNF production (**Figure 4C**). KEGG enrichment analysis suggested that immune-mediated signaling pathways, various human diseases including multiple virus infections, alcoholic liver disease, and autoimmune disease, and classical signaling pathways including the TNF signaling pathway, cytokine–cytokine receptor interaction, NF-kappa B signaling pathway, and MAPK signaling pathway were closely related to these immune microenvironment-related DEGs. These results demonstrated the crucial roles of immune microenvironment-related DEGs.

### Development and estimation of machine learning models

**Figure 5:**
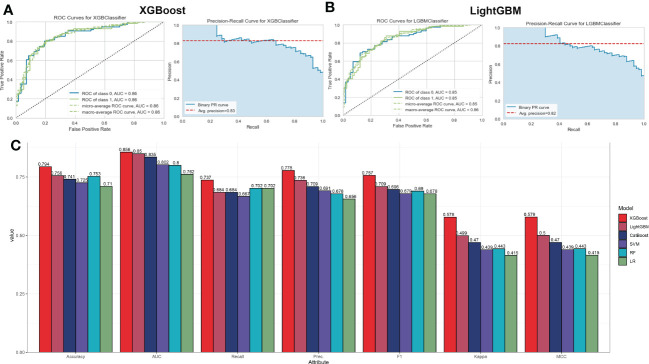
Evaluation the performance of the six machine learning algorithms in testing set. **(A, B)** The specific values of AUC and P-R in XGBoost **(A)** and LightGBM **(B)**, machine learning models. **(C)** Comprehensive estimate the performance of the six machine learning models including accuracy, recall, precision, F1, kappa, and MCC.

To ascertain the optimal machine learning model for predicting AD, we randomly split a total of 436 samples in the combined dataset (247 normal and 189 AD) into the training cohort (70%, N=305) and testing cohort (30%, N=131). The expression profiles of 31 immune microenvironment-related DEGs were selected as input variables, and six machine learning models, including XGBoost, CatBoost, SVM, LightGBM, LR, and RF were established to predict outcomes. The performance of multiple machine learning models (accuracy, AUC, recall, precision, F1, kappa, and MCC) in the training cohort was shown in **Figure S2A**. The LightGBM model displayed the highest accuracy (0.797), AUC (0.858), recall (0.736), precision (0.792), F1 (0.759), kappa (0.585), and MCC (0.591). Whereas the SVM model exhibited the lowest accuracy (0.731) and AUC (0.808). In the testing cohort, the XGBoost model acquired the best performance with an AUC value of 0.86 (CatBoost: 0.84, SVM: 0.80, LightGBM: 0.85, LR: 0.76, RF: 0.80) and a P-R value of 0.83 (CatBoost: 0.83, SVM: 0.77, LightGBM: 0.82, LR: 0.68, RF: 0.76) (**Figures S2A–E** and **Figures 5A–C**). To further estimate the performance of the six models in the testing cohort, the accuracy, recall, precision, F1, kappa, and MCC were also calculated and the results were presented in **Figure 5C**. Combined with these results, the XGBoost model is superior to other models, and the LightGBM model have the second-best performance. Therefore, the XGBoost and LightGBM models were selected for subsequent prediction.

### Global and local explanations of the machine learning models

**Figure 6:**
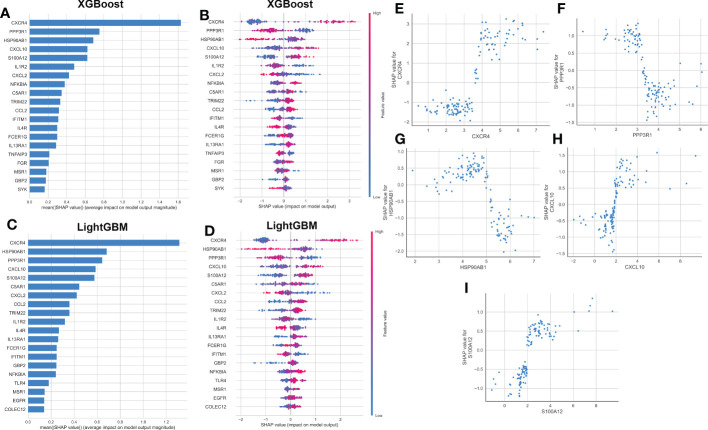
explanation of machine learning models using SHAP summary and dependence plot. **(A, B)** Importance matrix and SHAP summary plot showing the top 20 immune microenvironment-related genes contributing to the XGBoost model. **(C, D)** Importance matrix and SHAP summary plot showing the top 20 immune microenvironment-related genes contributing to the LightGBM model. Each line represented a feature variable, and the abscissa corresponded to the SHAP value. Red dots corresponded to higher feature values, and blue dots corresponded to lower feature values. **(E–I)** SHAP dependence plot showing the top 5 characteristic features shared by the XGBoost and LightGBM models. SHAP values more than zero represented a higher risk of AD.

To explain how the machine learning model worked in predicting AD onset, we aimed to open the ‘black box’ in the XGBoost and LightGBM models *via* SHAP values and elucidate the influence of each feature variable on the prediction model. The importance ranking of the feature variables based on the SHAP summary plot of the XGBoost model indicated that the top 5 most powerful variables contributing to the XGBoost model were CXCR4, PPP3R1, HSP90AB1, CXCL10, and S100A12 (**Figure 6A**). In addition, we employed SHAP dependency analysis to describe how a single characteristic variable influenced the outcome of the XGBoost predictive model (**Figure 6B**). The higher SHAP values of a characteristic variable, the more likely AD becomes. For example, in the XGBoost model, lower feature values of CXCR4 corresponded to negative SHAP values, which were closely related to a lower risk of AD onset. Contrast that with higher feature values of CXCR4 were corresponded to positive SHAP values and exerted a stronger impact on the prediction of AD onset. Furthermore, we also found the top 5 most important variables in the LightGBM model were consistent with those in the XGBoost model, with CXCR4, HSP90AB1, PPP3R1, CXCL10, and S100A12 being ranked in the top five (**Figure 6C**). The SHAP dependency analysis was also utilized to interpret the effects of each feature variable on the output of the LightGBM model (**Figure 6D**). More detailed information about the top 5 immune microenvironment-related DEGs affecting the outcome of the XGBoost model was presented in **Figures 6E–I**. In total, low expression levels of PPP3R1 and HSP90AB1 exerted a powerful influence on the development of AD. Additionally, high levels of CXCR4, CXCL10, and S100A12 were closely linked to AD progression.

**Figure 7:**
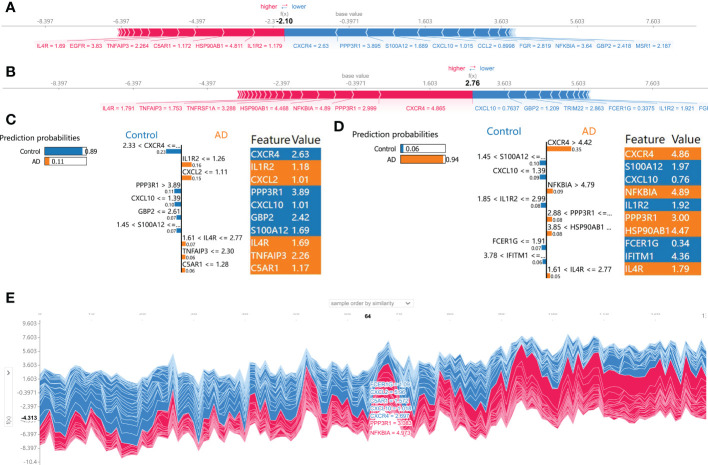
SHAP force plot and LIME algorithm for interpreting individual’s prediction results. **(A, B)** A normal individual **(A)** and AD case **(B)** were presented based on the SHAP force plot, respectively. The bars in red and blue corresponded to increased AD probability and decreased AD probability, respectively. **(C, D)** A normal individual **(C)** and AD case **(D)** were presented based on the LIME algorithm, respectively. The left side of the figure exhibited the results of the LIME predictions. The middle section lists exhibited the ten variables that have the greatest effects on normal or AD onset. The length of each feature bar indicated the importance of correspond feature in making predictions. The right panel exhibited the specific values ​​for the ten variables with the greatest effect on normal or AD onset. **(E)** The global explanation for all the normal and AD samples in the testing cohort were depicted based on the SHAP force plot.

Subsequently, the SHAP force plots and the LIME algorithm were employed to interpret the individualized prediction of AD *via* drawing the patient and normal subjects from the testing set. According to the SHAP force plots, numbers in bold corresponded to probabilistic predictions (f(x)), and the base values ​​represented predictions without model input. The blue bars on the right represented normal predictions and the pink bars on the left reflected predictions with increased AD probability. **Figures 7A, C** presented a normal case based on the SHAP force plot and LIME algorithm, respectively. The predicted AD probability based on the XGBoost model was 11%. Feature variables predicted by the XGBoost model that reinforce the onset of AD were IL1R2 expression of 1.18, CXCL2 expression of 1.01, IL4R expression of 1.69, TNFAIP3 expression of 2.26, and C5AR1 expression of 1.17. Feature variables that reduced the risk of AD included CXCR4, PPP3R1, CXCL10, GBP2, and S100A12. The predicted outcome of the XGBoost model for the current sample is the control, which is consistent with the actual outcome of the sample. Similarly, **Figures 7B, D** exhibited an AD case on the basis of the SHAP force plot and LIME algorithm, respectively. The predicted AD probability based on the XGBoost model was 94%. The patient’s elevated CXCR4 of 4.86, NFKBIA of 4.89, PPP3R1 of 3.00, HSP90AB1 of 4.47, IL4R of 1.79 resulted in increasing the AD risk, while the expression of S100A12 at 1.97, CXCL10 at 0.76, IL1R2 at 1.92, FCER1G at 0.34, and IFITM1 at 4.36 could lead to a lower risk of AD. The expected output of the XGBoost model is AD, and the actual result is also AD. **Figure 7E** depicted the global explanation for all the normal and AD brain tissue samples in the testing cohort.

### Selection and verification of characteristic genes

**Figure 8:**
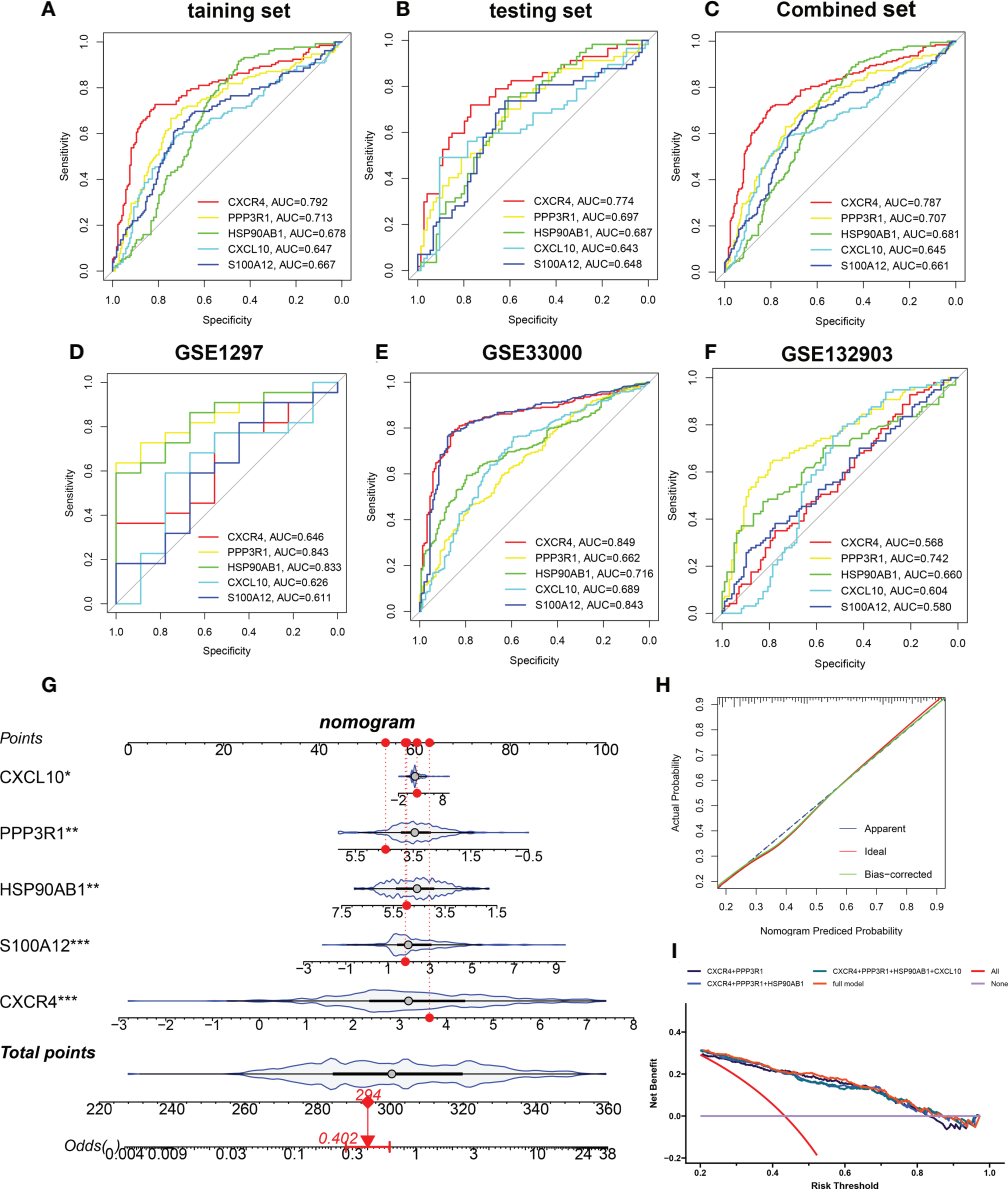
Validation of the diagnostic efficacy of characteristic genes. **(A–C)** ROC curves showing the diagnostic performance of characteristic genes in internal datasets including training set **(A)**, testing set **(B)**, and combined set **(C)**. **(D–F)** ROC curves showing the diagnostic performance of characteristic genes in external datasets including GSE1297 **(D)**, GSE33000 **(E)**, and GSE132903 **(F)**. **(G)** Representative nomogram showing the predicted risk for AD based on feature genes. **(H)** Representative calibration curve showing predicted performance of the nomogram. **(I)** DCA showing the clinical benefits of the nomogram. *p < 0.05, **p < 0.01, ***p < 0.001.

Based on the mean SHAP values, we have intersected the top 5 feature variables from the XGBoost and LightGBM predictive model and 5 characteristic genes (CXCR4, PPP3R1, HSP90AB1, CXCL10, and S100A12) shared by the XGBoost and LightGBM machine learning model were finally determined. Then, the diagnostic ability of each feature gene to predict AD progression in the internal datasets was assessed using ROC curve analysis. The AUC values of the ROC curves in the training set were 0.792 for CXCR4, 0.713 for PPP3R1, 0.678 for HSP90AB1, 0.647 for CXCL10, and 0.667 for S100A12 (**Figure 8A**). The AUC values of ROC curves in the testing set were 0.774 for CXCR4, 0.697 for PPP3R1, 0.687 for HSP90AB1, 0.643 for CXCL10, and 0.648 for S100A12 (**Figure 8B**). The AUC values of ROC curves in the combined set were 0.787 for CXCR4, 0.707 for PPP3R1, 0.681 for HSP90AB1, 0.645 for CXCL10, and 0.661 for S100A12 (**Figure 8C**). In addition, three external validation datasets were used: GSE1297 (CXCR4: AUC=0.646, PPP3R1: AUC=0.843, HSP90AB1: AUC=0.833, CXCL10: AUC=0.626, S100A12: AUC=0.611), GSE33000 (CXCR4: AUC=0.829, PPP3R1: AUC=0.662, HSP90AB1: AUC=0.716, CXCL10: AUC=0.689, S100A12: AUC=0.843), and GSE132903 (CXCR4: AUC=0.568, PPP3R1: AUC=0.742, HSP90AB1: AUC=0.660, CXCL10: AUC=0.604, S100A12: AUC=0.580) were utilized to further verify the diagnostic efficacy of these five characteristic genes (**Figures 8D–F**). Furthermore, an AD development prediction tool, the nomogram, was constructed by including these five signature genes associated with immune microenvironment. In the nomogram, the value of each signature variable is correlated with a score point, and the total scores were obtained *via* summing the scores of all feature variables, which represented the risk of AD onset (**Figure 8G**). The calibration curve confirmed the accuracy of the nomogram in diagnosing AD (**Figure 8H**). DCA revealed that the clinical application of the nomogram brought certain clinical benefits to AD patients (**Figure 8I**). Taking the above results together, we concluded that these feature genes associated with the immune microenvironment displayed better diagnostic ability for predicting the progression of AD.

### Identification of subtypes in the immune microenvironment of patients with AD

**Figure 9:**
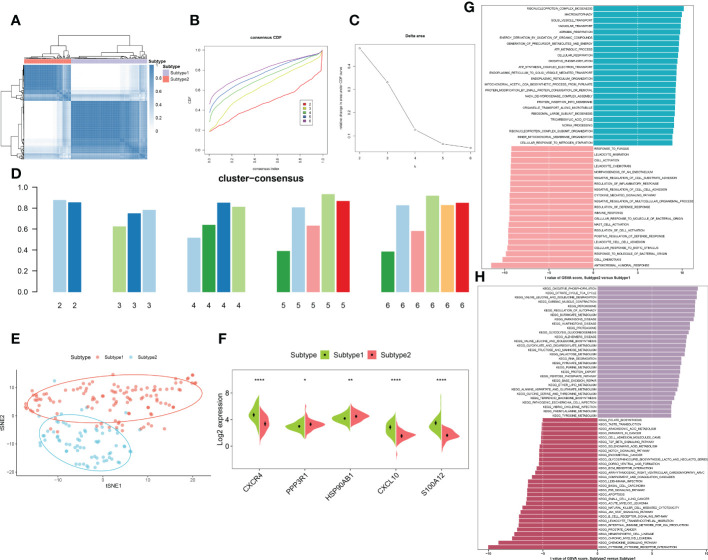
Identification and enrichment analysis of immune microenvironment subtypes. **(A)** Consensus clustering matrix when k = 2. **(B)** Consensus CDF curves when k=2 to 6. **(C)** Relative alterations in CDF delta area curves. **(D)** Consensus score of each subtype when k=2 to 6. **(E)** t-SNE diagram separated subtype1 (pink) and sybtype2 samples (blue). **(F)** Split violin plots revealing the expression of 5 characteristic genes between subtypes. **(G, H)** Differences in enriched biological functions **(G)** and hallmark pathways **(H)** between distinct immune microenvironment subtypes ranked by t values of GSVA scores. *p < 0.05, **p < 0.01, ****p < 0.0001.

To elucidate the immune microenvironment-related expression patterns in AD, we performed the consensus clustering algorithm to group the 189 AD brain tissue samples on the basis of 5 characteristic genes. The consensus matrix was regarded as the similarity matrix to determine the final subtypes. Based on the consensus clustering results, CDF plot, relative change of the CDF curve area, and the consistent cluster score, we selected k = 2 as the optimal value to group 189 patients into two different subtypes, with 112 samples in subtype1, and 77 samples in subtype2 (**Figures 9A–D**). tSNE analysis manifested the apparent difference between subtype1 and subtype2 (**Figure 9E**). As expected, there was eminent heterogeneity in the expression of 5 characteristic genes between these subtypes (**Figure 9F**). Differential analyses indicated that 609 upregulated and 1055 downregulated DEGs were identified between subtype1 and subtype2 (**Figure S3**).

We then characterized enriched biological functions and signaling pathways using gene sets from the MSigDB database, and performed GSVA to estimate the score of each patient. In immune microenvironment subtype1, immune response-related biological functions including mast cell activation, immune response, cell chemotaxis, cytokine-mediated signaling pathways, and inflammatory response (leukocyte cell migration, chemotaxis, and adhesion) were highly enriched. The biological functions of subtype2 were mainly involved in the transportation of vesicles, metabolic processes, oxidative phosphorylation, and the organization of the mitochondrial inner membrane (**Figure 9G**). Consistently, the pathways for immune microenvironment subtype1 enrichment were consistently linked to immune response, including the B cell receptor signaling pathway, cytokine-cytokine receptor interaction, production of intestinal IgA, the notch signaling pathway, and TGF-β signaling. Whereas in immune microenvironment subtype2, neurodegenerative diseases and metabolism-related pathways were activated (**Figure 9H**).

### Differentiation of immune characteristics between immune microenvironment subtypes

**Figure 10:**
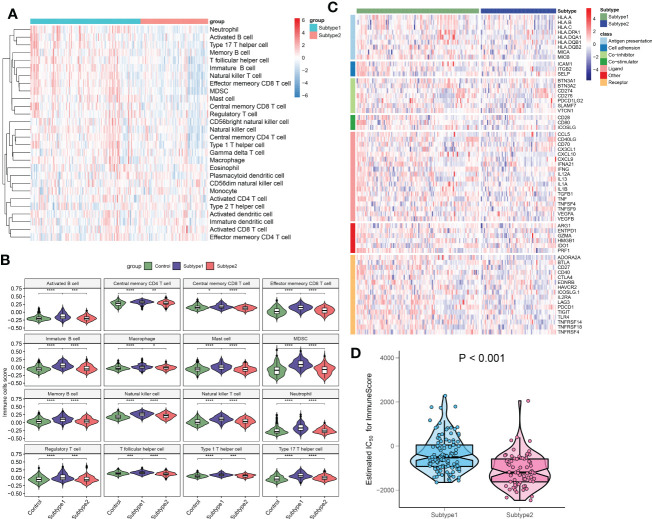
The immune characteristics of distinct immune microenvironment subtypes. **(A)** Heatmap showing the ssGSEA scores of 28 immune cell subpopulations between subtypes. **(B)** Violin diagram showing the differences of infiltrated immune cells among three groups. **(C)** Heatmap showing the differences in immune regulatory genes between subtypes. **(D)** Violin plots revealing the differences in immune scores between subtypes. *p < 0.05, **p < 0.01, ***p < 0.001, and ****p < 0.0001.

To better clarify and understand the biological and immunological differences and relationships between these immune microenvironment subtypes, we first compared the differences in 28 immune cell subsets within each subtype. A higher infiltration of T cells, including central memory CD4^+^ T cell, central memory CD8^+^ T cell, effector memory CD4^+^ T cell, natural killer T cell, regulatory T cell, type 1 T helper cell, and type 17 T helper cell were observed in patients with subtype1 when compared with subtype2 groups. Meanwhile, multiple B cells including activated B cell, immature B cell, and memory B cell, and innate immune cells including natural killer cell, macrophage, mast cell, MDSC, neutrophil also had higher enrichment scores in immune microenvironment subtype1 (**Figures 10A, B**). To look for the differences in immune characteristics between each subtype, we further evaluated the expression of immune regulatory genes in each subtype (**Figure 10C**). In immune microenvironment subtype1, all immune co-stimulator genes and nearly all immune genes related to antigen presentation and cell adhesion were consistently highly expressed. Meanwhile, immune microenvironment subtype1 also exhibited the enhanced expression of the immune co-inhibitor, ligand, receptor, and other associated genes (**Figures S4A–G**). Moreover, the immune scores from each immune microenvironment subtype were also compared, which represented a qualitative evaluation of immune characteristics. Patients with subtype1 had greater immune scores than the other subtype (**Figure 10D**). Combined with the above results, we eventually identified subtype1 as an immune subtype and subtype2 as a metabolism subtype.

### Correlation analysis and therapeutic target prediction

**Figure 11:**
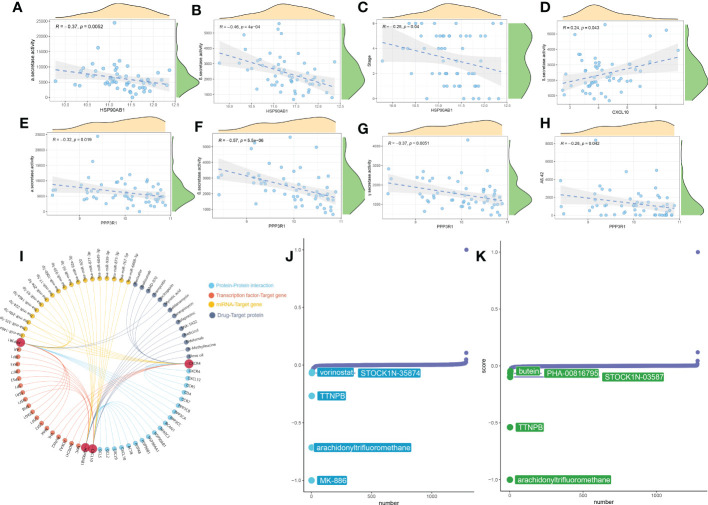
Correlation analysis and therapeutic target prediction. **(A–H)** Scatter plot showing the correlations between HSP90AB1, CXCL10, PPP3R1 and AD pathological markers (α-secretase activity, β-secretase activity, AD clinical stag, and Aβ-42 levels). **(I)** Prediction of upstream- or downstream- target molecules of 5 signature genes including protein-protein interaction, transcription factors, miRNA, and potential drugs. **(J, K)** CMap analysis revealing the potential therapeutic drugs for subtype1 **(J)** and subtype2 **(K)**, respectively.

To further explore the function of these 5 characteristic genes, we utilized the external dataset GSE106241, which included more detailed clinical information, to clarify the correlation between these characteristic genes and AD pathological biomarkers. We observed that HSP90AB1 was negatively correlated with α-secretase activity (R= -0.37), β-secretase activity (R= -0.46), and AD clinical stage (R= -0.28) (**Figures 11A–C**). Whereas CXCL10 was positively associated with β-secretase activity (R= -0.46) (**Figure 11D**). Meanwhile, PPP3R1 also exhibited negatively correlated with the activity of α-secretase (R= -0.32), β-secretase (R= -0.57), γ-secretase (R= -0.37), and Aβ-42 levels (R= -0.28) (**Figures 11E–H**). Furthermore, **Figure 11I** depicts additional information about these five characteristic genes, such as protein-protein interactions, predicted transcription factors, miRNA, and multiple drugs targeting these five characteristic genes. Finally, we explore the potential therapeutic drugs targeting immune subtype1 and metabolism subtype2 using the CMap analysis. The lower the CMap score for a small molecule compound, the more likely it is to have the ability to treat the disease. MK-866, arachidonyltrifluoromethane, TTNPB, vorinostat, and STOCKIN-3584 were the top five small-molecule compounds with the lowest CMap score in subtype1 (**Figure 11J**), whereas TTNPB, butein, PHA-00816795, and STOCKIN-3584 were metabolism subtype2-related small-molecule compounds (**Figure 11K**). Subtype1 and subtype2 shared three common small molecular compounds, including arachidonyltrifluoromethane, TTNPB, and STOCKIN-3584. The CMap score indicated that MK-866 and arachidonyltrifluoromethane were the most suitable therapeutic drugs for targeting immune subtype1 and metabolism subtype2, respectively.

### External validation of differential expression of characteristic genes

**Figure 12:**
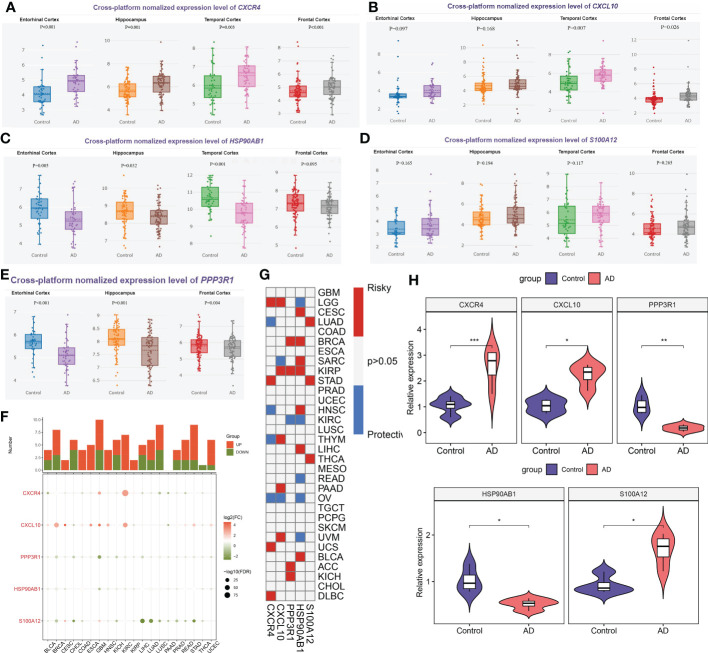
External validation of characteristic genes. **(A–E)** AlzData database showing the expression levels of CXCR4 **(A)**, CXCL10 **(B)**, HSP90AB1 **(C)**, S100A12 **(D)** and PPP3R1 **(E)** in different brain regins. **(F)** Histogram (upper panel) showing the amount of up-regulated or down-regulated DEGs, and the heatmap showing the fold change and FDR of 5 characteristic genes in each cancer. Red color corresponding to up-regulation and green color corresponding to down-regulation. **(G)** Summary of the correlation between 5 characteristic genes and survival of cancer patients. Red color corresponding to a higher expression of characteristic gene related with worse survival, while blue color corresponding to an correlation with better survival. Only p-value less than 0.05 was exhibited. **(H)** Violin plots revealing the expressional differences in CXCR4, CXCL10, PPP3R1, HSP90AB1, and S100A12 between control and AD groups. *p < 0.05, **p < 0.01, ***p < 0.001.

Firstly, we employed the online tool AlzData database to elucidate the expression landscape of characteristic genes in different brain regions of AD patients. The expression of CXCR4 was notably increased, while HSP90AB1 and PPP3R1 were markedly lower in the hippocampus and cortex tissues of AD patients relative to normal controls. In addition, a significant increment of CXCL10 expression was also observed in the temporal cortex and frontal cortex of patients with AD. However, the difference in the expression of S100A12 in different brain regions between normal subjects and AD patients was not significant (**Figures 12A–E**). Subsequently, we perform pan-cancer analysis to investigate the differential expression of these five hallmark genes between 20 cancer types and adjacent normal tissues. It was worth noting that the expression levels of CXCR4, PPP3R1, HSP90AB1, CXCL10, and S100A12 were substantially elevated in multiple types of cancer tissues (**Figure 12F**). Next, we aimed to focus on those signature genes that were notably associated with patient survival in 33 cancer types. We found that all of the signature genes were closely related to the overall survival of patients with at least three cancer types (**Figure 12G**). Finally, we performed the RT-PCR analysis to further verify the expression landscape of these five characteristic immune microenvironment-related genes. Similar to the results of datasets in brain tissue samples, the expression levels of CXCR4, CXCL10, and S100A12 were notably higher in AD cortical neurons, while HSP90AB1 and PPP3R1 genes exhibited significant downregulation (**Figure 12H**).

## Discussion

The creation of a clinically viable and useful classifier to direct AD treatment is crucial given the considerable variation in outcome observed in AD patients. We discovered that the immune microenvironment status was closely associated to the beginning and development of AD in patients. Then, using six machine learning methods, including LightGBM, CatBoost, XGBoost, RF, LR, and SVM, we identified 5 immune microenvironment-related characteristic genes that were strongly correlated with AD pathology biomarkers and capable of reliably forecasting the course of AD. The output of machine learning models was interpreted using the SHAP and LIME algorithms. Additionally, based on these distinguishing genes, we discovered two distinct immune microenvironment subtypes, each of which displayed distinct enriched functions and pathways, immune cell infiltration, immunological features, and therapeutic drugs. These results offer a fresh perspective on the relationship between the immune microenvironment in the brain tissues and the prognosis and classification of AD patients.

It was generally recognized that both innate and adaptive immune responses would decline with age. Therefore, aging negatively impacts immunity and may increase the risk of various autoimmunity and inflammation diseases (38). Several researchers have reported the role of the immune microenvironment in patients with AD (6). It was found that the dysregulation of both central and peripheral immune responses is regarded as the basis of AD pathogenesis and progression. On one hand, an imbalance in peripheral and central T-cell immunity has been observed in AD patients, as evidenced by increased proportions of central and effector memory T cells (39, 40), suggesting that the alterations in the distribution and activity of functional subpopulations of peripheral lymphocytes may be a critical pathogenic mechanism causing disease progression. Other studies have proven that the higher proportion of CD4^+^ T cells in AD patients mainly includes type17 and type 19 T help cells (41, 42), and regulatory T cells-mediated immunomodulation is also involved in AD pathology (43). On the other hand, excessive accumulation of cerebral Aβ peptides persistently activates the microglia- and astrocyte-dependent immune cascade signaling, and the prolonged neuroinflammatory conditions can lead to the infiltration of innate immune cells in the central nervous system (CNS), ultimately contributing to disease progression, neuronal dysfunction and damage. Studies of the mouse models of Aβ pathology and AD patients have observed the significant infiltration of peripheral innate immune cells including macrophages, natural killer cells, and neutrophils in the CNS (11, 44, 45). The depletion or inhibition of neutrophils could exert neuroprotective effects *via* reducing Aβ neuropathology and improving memory (11). As an essential component of the innate immune system and a member of cytotoxic lymphocytes NK cells are capable of rapidly killing cells *via* cytotoxic granules-mediated apoptosis and inflammatory factors such as INF-γ, as well as stimulating the activation of other immune cells to cause an immune cascade response when stimulated (46, 47). The cytotoxic activity of peripheral blood NK cells is declining with age, and the infiltration levels of peripheral NK cells were significantly higher in the brains of AD humans and mice (48–50). Additionally, Genome-wide association studies have revealed the close correlation between multiple immune-related genes and the pathology of AD. For example, homeostatic dysregulation of microglia (disease-associated microglia) could be observed in AD brain, as evidenced by the accumulation of the phagocytic receptor TREM2-mediated phagocytic and lipid metabolism genes (51, 52). Another phagocytic receptor, CD33, acts as the upstream regulator of TREM2, and studies have confirmed that the crosstalk between CD33 and TREM2 promotes the pathogenesis of AD *via* regulating the IL-1β/IL-1RN singling pathway (53). Consistently, in our current study, we identified six most correlated immune cells, including plasmacytoid dendritic cell, type 17 T helper cell, immature B cell, NK cells, MDSC, and neutrophil based on the LASSO regression algorithm (**Figure 2**). The infiltrated immune cells subtypes revealed that both activations of innate and adaptive immunity were observed in AD patients, thus presenting a poorer prognosis. Targeting these immune cells may provide a theoretical basis for the treatment of AD. Meanwhile, we have identified 31 DEGs that were most associated with the above six immune cell subsets, most of which exhibited strong synergistic effects. Functional enrichment analysis suggested that these DEGs were notably enriched in immune-related functions and pathways (**Figures 3**, **4**), suggesting that the progression of AD might be a result of the combination of multiple immune genes and cells.

Accurate early prediction and diagnosis of AD are required for the timely identification of patients at high risk of AD so that preventive approaches can be developed in a timely manner. Recently, due to the excellent performance in clinical diagnosis, various machine learning algorithms have been widely utilized to predict new biomarkers and obtain new information about disease pathogenesis (54, 55). Machine learning not only provides an unbiased approach to predict patient’s clinical status, but also can detect previously unknown conditions and identify new biomarkers (56, 57). In our current study, of the six machine learning algorithms we tested, we observed that an XGBoost approach exhibited the best performance, as evidenced by the highest AUC, P-R curve area, accuracy, recall, precision, F1, kappa, and MCC identifying AD patients (**Figure 5A**). The LightGBM algorithm performed second only to the XGBoost model (**Figure 5B**). The summary of feature importance in both the XGBoost and LightGBM models suggested that CXCR4 was the most critical factor contributing to the pathogenesis of AD patients (**Figures 6A, B**). CXCR4 is an evolutionarily highly conserved member of the GPCR family. As the major receptor for CXCL12, CXCR4 is widely enriched in CNS and plays a critical role in regulating neurotransmission, synaptic plasticity, and glial interactions (58, 59). Several studies have demonstrated that there is a powerful correlation between the dysregulated CXCR4 and neurodegenerative diseases including AD, and the inhibition of CXCR4/CXCL12 signaling pathways is able to alleviate glutamate release mediated toxic cascade and neuronal apoptosis (60, 61). Meanwhile, according to the XGBoost and LightGBM models, other four vital feature variables were PPP3R1, HSP90AB1, CXCL10, and S100A12 (**Figures 6A,B**). Bioinformatics analysis indicated that the downregulation of PPP3R1 can serve as novel biomarkers for patients with AD and the potential mechanisms of low PPP3R1 involved in AD pathogenesis mainly involve in axon guidance, glutamatergic-mediated synapses transport, LTP, and MAPK signaling pathway (62). HSP90AB1, which is a chaperone of Hsp90 family that is closely linked to astrocytes, was reported to be a neuroprotective factor in AD patients *via* reducing the accumulation of cerebral Aβ and tau (63). As a vital chemokine, a higher concentration of CXCR10 was observed in the brain tissues of AD animal models, indicating its pathogenic role in contributing to AD progression (64). S100A12 is a novel inflammation-related protein expressed by neutrophils and can be induced in many inflammatory cells. It has been demonstrated that the abnormal expression of S100A12 in brain samples could aggravate AD-induced inflammatory insult (65). These findings were consistent with the results in our study that the overexpression of CXCR4, CXCL10, and S100A12, and the decreased PPP3R1 and HSP90AB1 might be predictive for poor prognosis of AD patients. Furthermore, the external validation datasets, constructed nomogram, calibration curve, and DCA demonstrated the satisfactory diagnostic ability of these 5 characteristic genes (**Figure 8**).

Most of the previous studies on AD are based on single or several types of immune cells and lack a comprehensive assessment of the immune profile of AD patients (6, 66–69). Our research comprehensively explored 28 immune cell subsets and various immune-modulators in the AD immune microenvironment, and based on the advantages of machine learning in clinical applications, we finally identified six key immune cell subtypes (plasmacytoid dendritic cell, type 17 T helper cell, immature B cell, natural killer cell, MDSC, and neutrophil) and five vital immune genes (CXCR4, PPP3R1, HSP90AB1, CXCL10, and S100A12) that can accurately predict AD progression, some of which have never been reported in AD before. In addition, recent studies only depict the expression landscape of immune cell subsets or immune-related genes based on small sample sizes, and lack more in-depth studies (50, 70, 71). What’s more, the immune-related molecular subtypes in AD patients also remain unknown and need further clarification. In this study, we combined several datasets and evaluated the immune characteristics of AD patients with a larger sample size, and performed an external validation with several independent datasets, which makes our study more complete and credible. Additionally, we classified the AD patients into two distinct subtypes based on the immune microenvironment-related feature genes (**Figures 9A–E**). We observed that subtype1 was mainly enriched in immune response-related functions and pathways, while subtype2 was associated with metabolism (**Figures 9G, H**). Meanwhile, subtype1 exhibited a higher infiltration of immune cells and immune scores. Intriguingly, the immune characteristics of subtype1 were even higher, as evidenced by the enhanced expression of co-stimulator, cell adhesion, co-inhibitor, ligand, and receptor associated immune genes (**Figures 10A–D**). Therefore, we recognized subtype1 as an immune subtype and subtype2 as a metabolism subtype. These findings also show that dysregulation of metabolism and the immune microenvironment may be defining features of AD subtypes as well as critical pathology mechanisms contributing to AD heterogeneity. Moreover, the potential small-molecular compounds of immune subtype1 and metabolism subtype2 were screened, respectively. We finally identified MK-866 and arachidonyltrifluoromethane as the most suitable target drugs for immune subtype1 and metabolism subtype2, respectively (**Figures 11J, K**).

However, there are a few issues with the current study that need to be clarified. First off, as this work was based on publicly available datasets, additional prospective samples for experimental evaluation are required for additional validation. Second, the samples employed for the identification of molecular subtypes or for the prediction of machine learning algorithms were somewhat tiny, necessitating a higher AD sample size for validation. Finally, it is impossible to fully evaluate the distinct subtypes of AD patients due to the lack of information on crucial clinical characteristics such as survival time, disease stage, responsiveness to medication, smoking, drinking, and previous therapies.

## Conclusion

The tight relationship between immune microenvironment state and AD pathogenesis was thoroughly explained by our study. Additionally, we discovered five distinct immune microenvironment-related genes (CXCR4, PPP3R1, HSP90AB1, CXCL10, and S100A12) that, when combined with interpretable machine learning methods, could successfully forecast the development of AD. Additionally, we put up a brand-new molecular classification for AD that includes immunological and metabolic subtypes. Together, a thorough reflection of each individual AD’s immune microenvironment pattern helps us to better understand the etiology of AD, offers novel diagnostic clues, and eventually creates a potential strategy for treating AD on an individual basis.

## Data availability statement

The original contributions presented in the study are included in the article/**Supplementary Material**. Further inquiries can be directed to the corresponding authors.

## Ethics statement

The animal study was reviewed and approved by The Institutional Animal Care and Use Committee of Fujian Medical University.

## Author contributions

JC and MZ conceived and designed the study. YL, PL, CL, XL, and LW collected the original data and performed the analysis. YL and PL accomplished the initial manuscript. FL, CL, and XL searched related literatures. MC performed the RT-PCR analysis. JC, MZ, and FL reviewed and edited the paper. All authors contributed to the article and approved the submitted version.

## Supplementary material

Supplementary Figure 1 Principal component analysis (PCA). **(A, B)** PCA of GSE48350, GSE5281 and GSE28146 datasets before **(A)** and after **(B)** batch correlation. The colors represented samples from three different datasets, respectively.

Supplementary Figure 2 Comprehensive estimate the performance of the six machine learning models in the training set. **(A)** Comparison of diagnostic efficacy of six machine learning models in the training set. **(B–E)** The specific values of AUC and P-R in CatBoost **(B)**, SVM **(C)**, LR **(D)**, and RF **(E)** machine learning models in the test set.

Supplementary Figure 3 Differential gene screening between subtype1 and subtype2. **(A, B)** Heatmap **(A)** and volcano plot **(B)** exhibiting the expression profile of up-regulated and down-regulated DEGs.

Supplementary Figure 4 The expression profiles of immune-modulators and immune checkpoints. **(A–G)** Split violin plots revealing the expression of immune regulatory genes associated with antigen presentation **(A)**, cell adhension **(B)**, co-inhibitor **(C)**, co-inhibitor **(D)**, ligand **(E)**, other **(F)**, and receptor **(G)**. **p* < 0.05, ***p* < 0.01, ****p* < 0.001, *****p* < 0.0001, ns, no significance.
