# Supplementary figures and images for: Identification of immune microenvironment subtypes and signature genes for Alzheimer’s disease diagnosis and risk prediction based on explainable machine learning
Source: Front Immunol. 2022 Dec 8;13:1046410. doi: 10.3389/fimmu.2022.1046410 (PMC9773397; doi:10.3389/fimmu.2022.1046410)

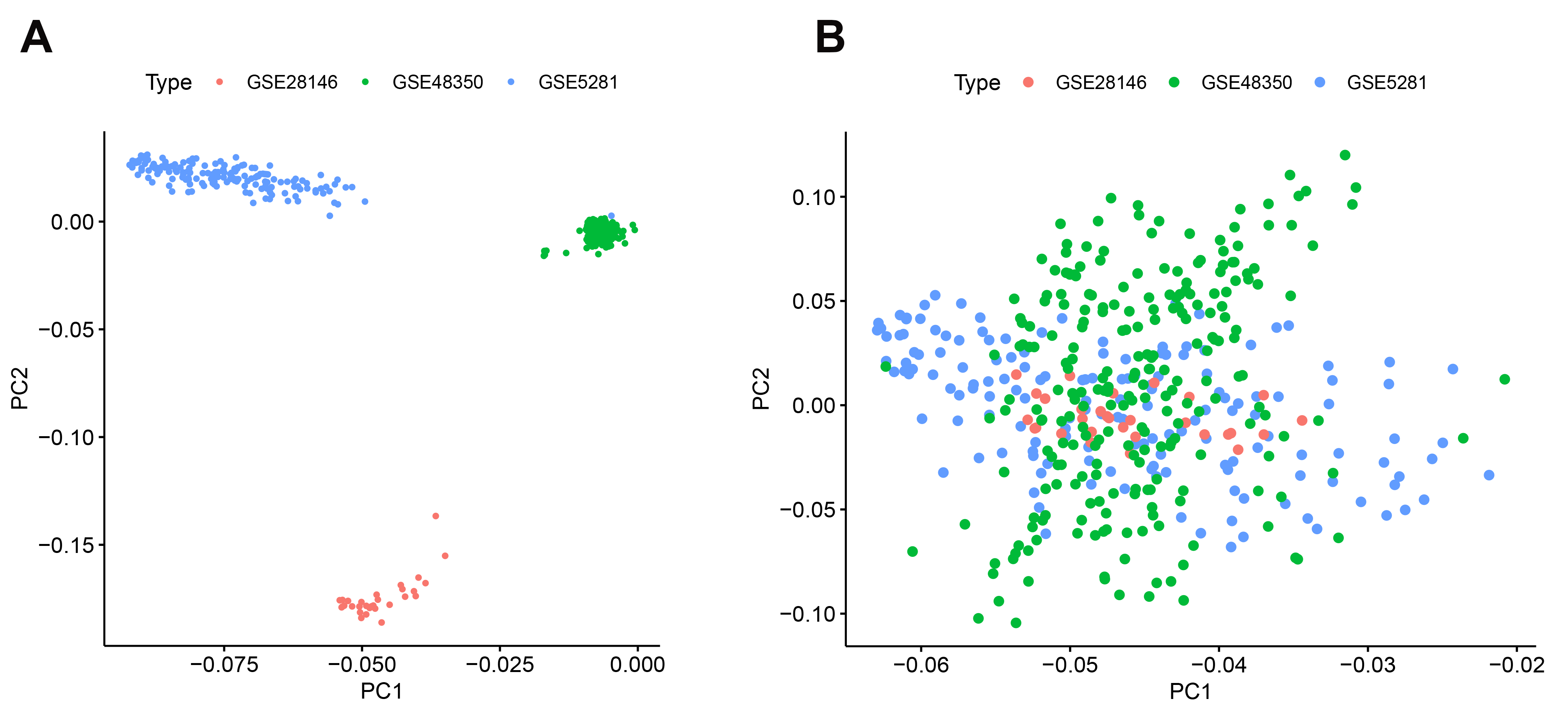

Supplement: Supplementary Figure 1 — Principal component analysis (PCA). (A, B) PCA of GSE48350, GSE5281 and GSE28146 datasets before (A) and after (B) batch correlation. The colors represented samples from three different datasets, respectively. [file Image_1.tif]

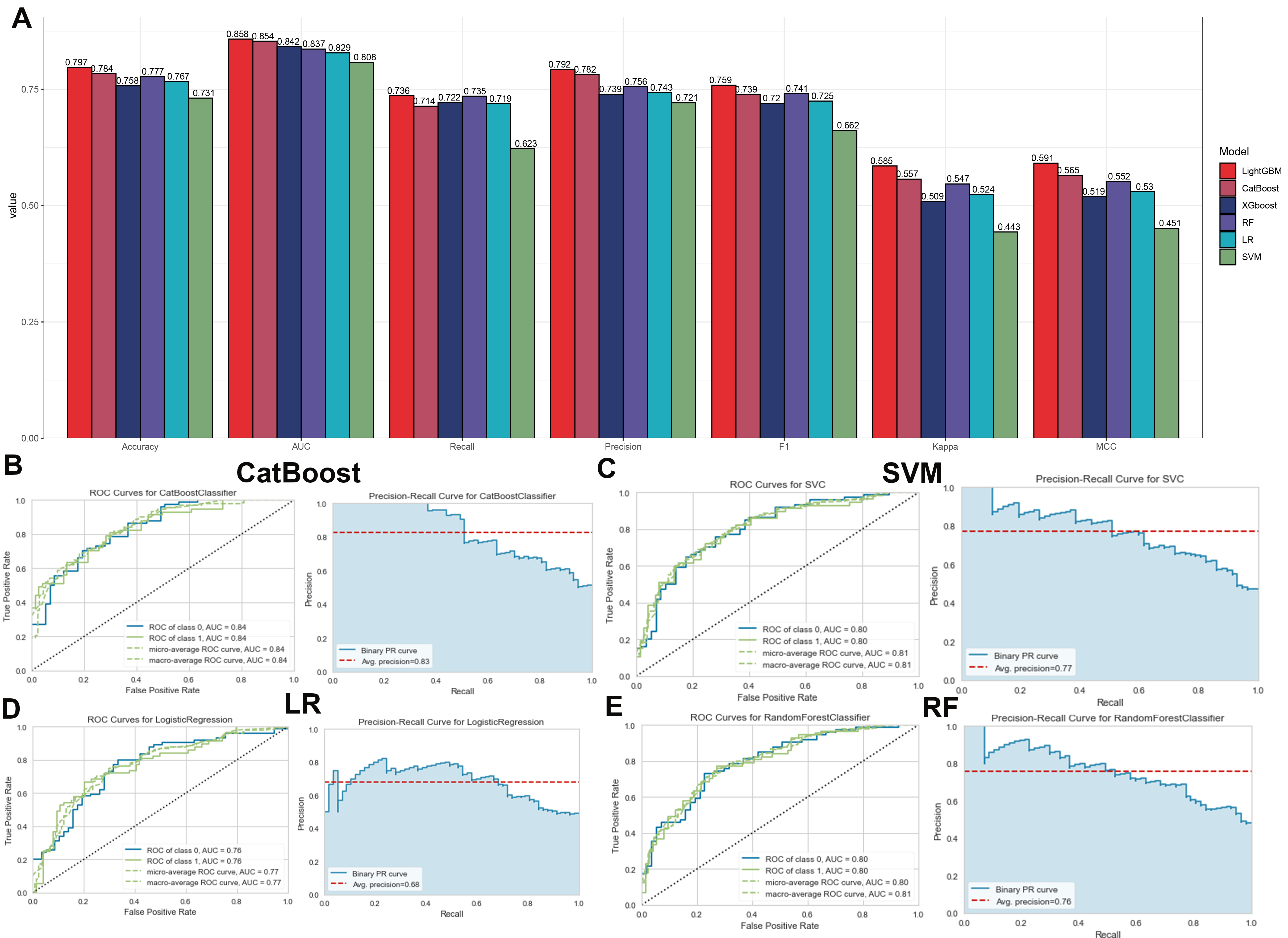

Supplement: Supplementary Figure 2 — Comprehensive estimate the performance of the six machine learning models in the training set. (A) Comparison of diagnostic efficacy of six machine learning models in the training set. (B–E) The specific values of AUC and P-R in CatBoost (B), SVM (C), LR (D), and RF (E) machine learning models in the test set. [file Image_2.tif]

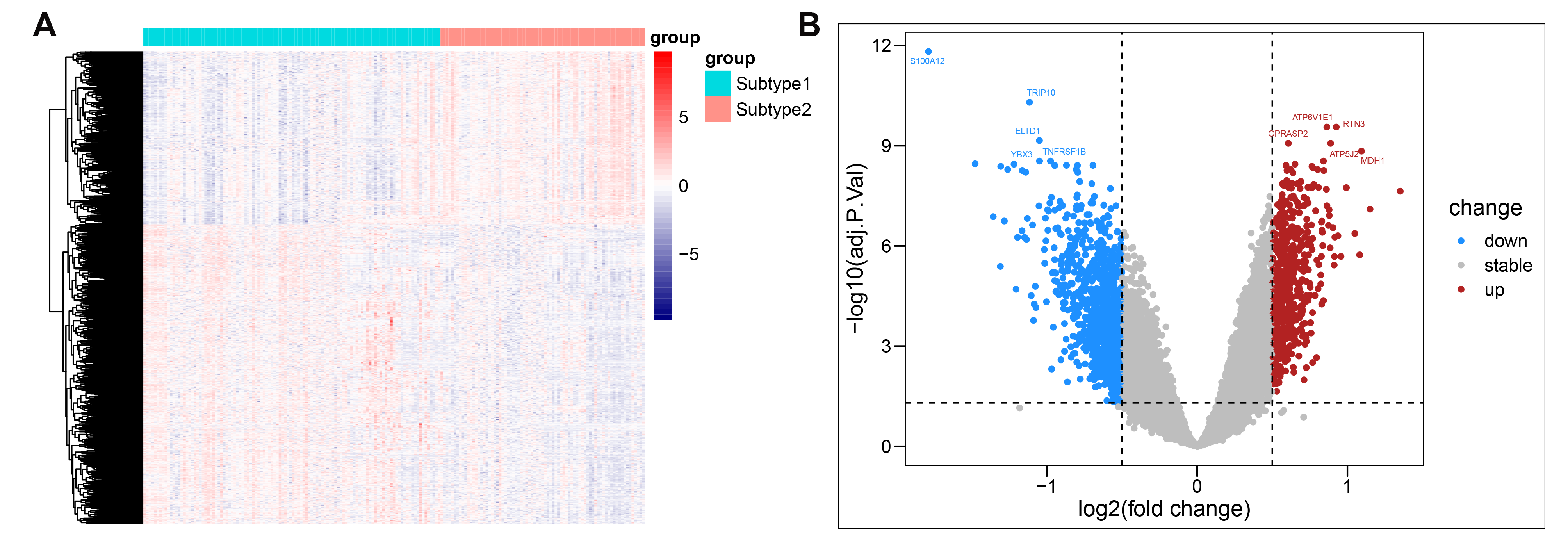

Supplement: Supplementary Figure 3 — Differential gene screening between subtype1 and subtype2. (A, B) Heatmap (A) and volcano plot (B) exhibiting the expression profile of up-regulated and down-regulated DEGs. [file Image_3.tif]

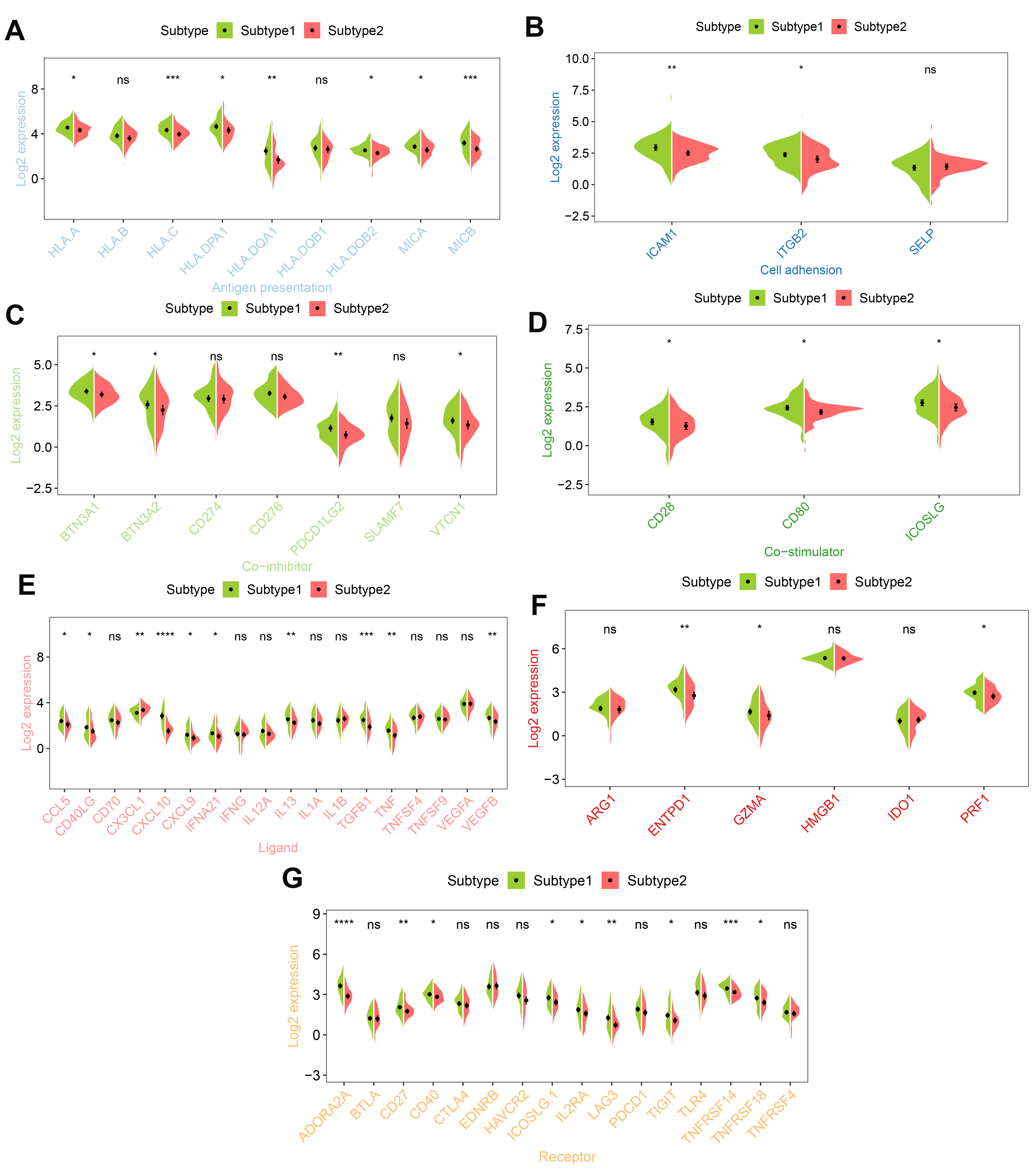

Supplement: Supplementary Figure 4 — The expression profiles of immune-modulators and immune checkpoints. (A–G) Split violin plots revealing the expression of immune regulatory genes associated with antigen presentation (A), cell adhension (B), co-inhibitor (C), co-inhibitor (D), ligand (E), other (F), and receptor (G). *p < 0.05, **p < 0.01, ***p < 0.001, ****p < 0.0001, ns, no significance. [file Image_4.tif]
